# Detection of *Cryptosporidium parvum* in a Red-Eared Slider Turtle (*Trachemys scripta elegans*), a Noted Invasive Alien Species, Captured in a Rural Aquatic Ecosystem in Eastern Poland

**DOI:** 10.2478/s11686-020-00180-8

**Published:** 2020-03-05

**Authors:** Artur Rzeżutka, Agnieszka Kaupke, Bartłomiej Gorzkowski

**Affiliations:** 1 Department of Food and Environmental Virology, National Veterinary Research Institute, Al. Partyzantów 57, 24-100 Puławy, Poland; 2 Epicrates Foundation, ul. Orlika Ruckemana 12/30, 20-244 Lublin, Poland

**Keywords:** *Cryptosporidium parvum*, Detection, Red-eared slider turtle, Invasive species

## Abstract

**Purpose:**

Little is known about cryptosporidiosis in turtles of invasive alien species (IAS) inhabiting European bodies of fresh water. In this article, we report an occurrence of *Cryptosporidium parvum* in a red-eared slider turtle (*Trachemys scripta elegans*) captured in a rural aquatic ecosystem in eastern Poland.

**Methods:**

A pair of samples consisting of feces and scrapings of intestinal mucosa (taken during necropsy) were collected from 104 animals representing the four IAS turtle species red-eared slider, yellow-bellied slider, false map and Cumberland slider. The animals were trapped in running and standing freshwater ecosystems across the Lublin province. Parasite genomic DNA was extracted from samples using a modified alkali wash and a heat-lysis method and identification of the *Cryptosporidium* species was performed at the 18SSU rRNA and COWP loci.

**Results:**

The presence of *Cryptosporidium* DNA was only detected in one sample of intestinal scraping collected from a red-eared slider. A phylogenetic analysis of a 18SSU rRNA gene fragment showed 100% sequence identity between the *C. parvum* strain isolated from the turtle and other *C. parvum* strains previously detected in cattle from the Lublin province.

**Conclusions:**

There was no clinical evidence that the red-eared slider turtle was truly infected rather than being merely a mechanical parasite carrier. Sporadic detection of this protozoan parasite in the studied population of IAS turtles could be associated with low natural occurrence of *Cryptosporidium* infections in this animal species. The results provide evidence for possible transmission of zoonotic *Cryptosporidium* species by IAS turtles.

## Introduction

Infections caused by *Cryptosporidium* protozoan parasites were recognised in more than 150 animal species. The group of vertebrates in which *Cryptosporidium* infections are poorly known are reptiles, with infections usually following an asymptomatic course [1, 2]. Cryptosporidiosis in tortoises mainly occurred among animals kept in poor conditions in captivity [3, 4] or suffering from viral or bacterial diseases [5, 6]. In tortoises, *Cryptosporidium testudinis* (*Cryptosporidium* tortoise genotype I), *C*. *ducismarci* (genotype II) and III are the genotypes mainly responsible for infections [2, 4]. Although *Cryptosporidium* infections have also been reported in turtles, the identification of the parasite at species level has not been performed [7, 8]. Similarly to tortoises, turtles are very popular as companion animals. It is reported that 690,401 turtles representing 15 species were imported to Europe in the years 2008–2012 [9]. The major varieties kept as pets are red-eared sliders (*Trachemys scripta elegans*), common map turtles (*Graptemys geographica*), painted turtles (*Chrysemys picta*), and common snapping turtles (*Chelydra serpentina*) [9]. They are alien species in Europe and have been released without sanction; only thereby are they found in the wild on this continent. Their presence in natural environments is undesirable and as invasive species they pose threats to local ecosystems [10]. Additionally, they could be a source of pathogenic microorganisms for native turtle species but also zoonotic pathogens for humans [11]. In this article, we report an occurrence of *C. parvum* in a captured turtle of an invasive alien species (IAS) from a rural aquatic ecosystem.

## Material and Methods

**Fig. 1:**
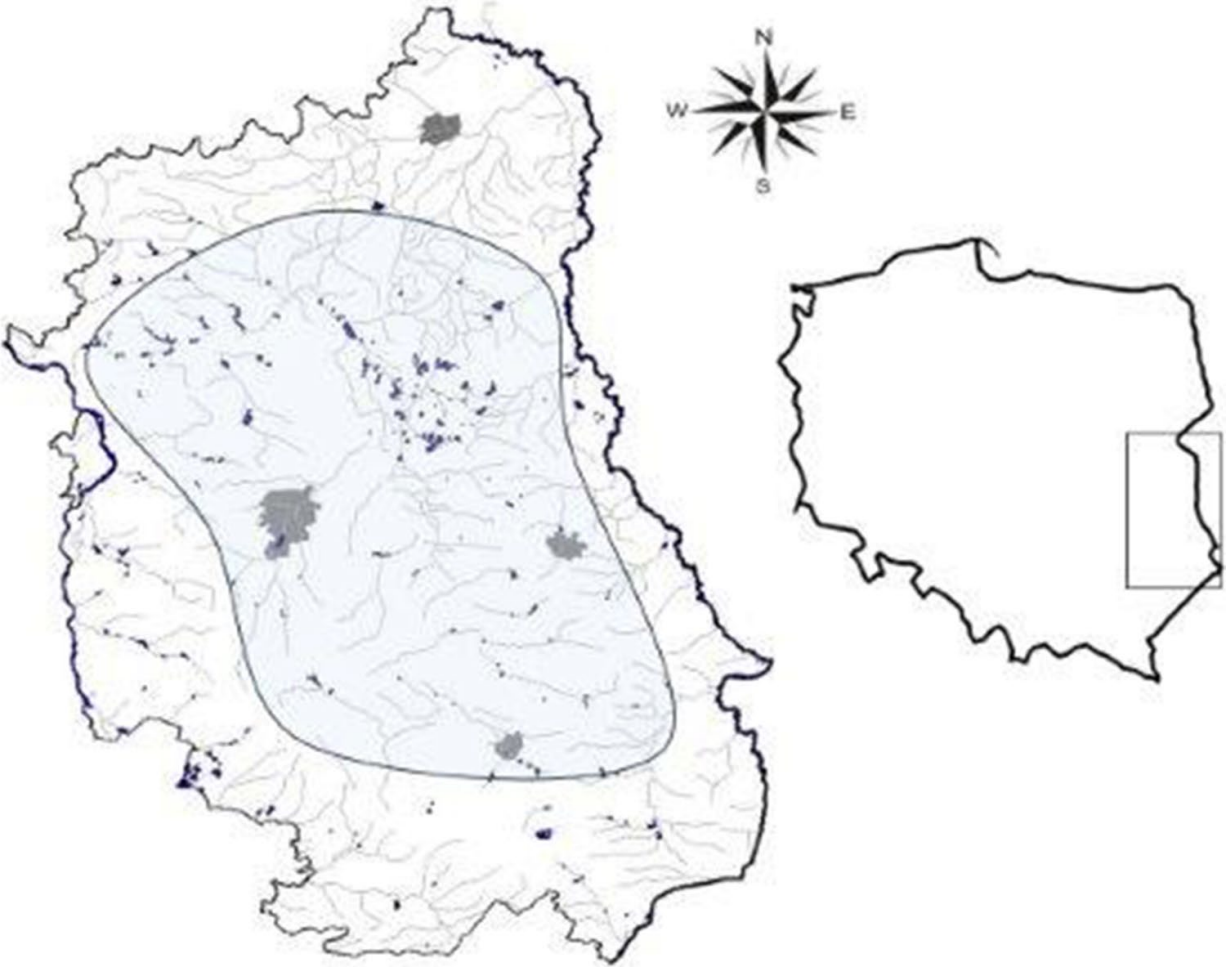
Location of turtle capture sites in the Lublin province

Fecal samples and/or scrapings of intestinal mucosa were collected from 104 turtles belonging to the following species: red-eared slider (*Trachemys scripta elegans*), yellow-bellied slider (*Trachemys scripta scripta*), false map turtle (*Graptemys pseudogeographica*), and Cumberland slider (*Trachemys scripta troostii*). For the majority of animals (67 turtles), a pair of samples (feces and intestinal scrapings) was taken for testing. In the case of 37 animals only intestinal scrapings were collected due to difficulties in obtaining fecal material. Feces was taken after trapping, during transportation or at the beginning of quarantine (stress associated defecation). For quarantined animals defecation was occasionally observed and voided feces quickly dissolved in water present in a terrarium. Therefore renewed sampling of the feces was not possible. IAS turtles were found inhabiting running and standing freshwater ecosystems across the Lublin province and were captured over a 3-year period from 2015 to 2017 (Fig. 1). Immediately after trapping, their health status and condition were assessed. Subsequently animals were individually placed in a terrarium for 14 days’ quarantine which allowed fecal material to be collected. After quarantine, turtles were subjected to euthanasia and necropsy. Detailed information on the animal trapping sites, species and number of collected turtles are presented in Table 1.

**Table 1:** Information on the animal trapping sites, species and number of collected turtles

District	Waterbody	Turtle species	Number of animals
Włodawa	Wytyckie Lake	*T. scripta scripta*	(2^b^)
		*T. scripta elegans*	2
	Białe Lake		1
Świdnik	Nd	*T. scripta elegans*	1
Łęczna	Piaseczno Lake	*T. scripta elegans*	2
		*T. scripta scripta*	1
	Nd	*T. scripta scripta*	1
		*T. scripta elegans*	1
	Uściwierz Lake	*T. scripta elegans*	1
	Zagłębocze Lake		2
		*T. scripta elegans*	1
Lublin	Zęmborzyce Lake	*T. scripta scripta*	17 (1^b^)
		*T. scripta elegans*	25 (1^a,b^)
		*T. scripta troostii*	7
	City pond	*T. scripta elegans*	1
	Nd		3
		*T. scripta elegans*	4
	Bystrzyca river	*T. scripta elegans*	1
	Ciemięga river	*T. scripta scripta*	1
Zamość	City pond	*T. scripta elegans*	3
		*T. scripta scripta*	1
Lubartów	Nd	*T. scripta elegans*	3
	Firlej Lake		2
Chełm	Nd	*T. scripta elegans*	1
	Janówka river, left tributary of Uherka		(1^b^)
	Wieprz river		1
Puławy	City pond	*T. scripta elegans*	7(1^b^)
Krasnystaw	Nd	*T. scripta scripta*	3
		*T. scripta elegans*	1
Zwoleń	Nd	*Graptemys pseudogographica*	1
Warszawa	Nd	*T. scripta troostii*	1
		*T. scripta scripta*	1
Rzeszów	Wisłoka river	*T. scripta scripta*	1
Wrocław	City pond	*T. scripta elegans*	1
Gdynia	Nd	*T. scripta elegans*	1
Krosno	City pond	*T. scripta elegans*	1

Numbers in brackets indicate number of diseased turtles of the given species which were captured in the particular waterbody

Nd - no data

^a^Animal with signs of disease determined on the basis of physical examination

^b^Animals with organ lesions observed during necroscopy

Parasite genomic DNA was extracted from 0.1 g of feces or intestinal scrapings using a previously described method of an alkali-wash and heat-lysis developed by Millar et al. [12] with further modifications [13]. A positive extraction control (turtle feces contaminated with *C*. *parvum* oocysts (Iowa strain, Waterborne, Inc., New Orleans, LA, USA), and a negative control (water instead of the analyzed template) were included and simultaneously processed for each set of samples. To improve DNA quality and purity it was purified further with a GeneMATRIX PCR/DNA Clean-Up Purification Kit (EURx, Ltd., Gdańsk, Poland) as recommended by the manufacturer. The identification of *Cryptosporidium* species was performed at the 18SSU rRNA and COWP loci [14, 15]. Subsequently a restriction fragment length polymorphism (RFLP) analysis was performed for the 18SSU rRNA-positive product as previously described [15, 16]. A definitive identification of detected *Cryptosporidium* species was performed based on sequence analysis of the 18SSU rRNA amplicon [13]. The obtained nucleotide sequence was compared to other *Cryptosporidium* sequences available in the GenBank using a neighbor-joining phylogenetic tree with Kimura two-parameter model (MEGA 7.0.9). The reliability assessment of a phylogenetic tree topology (bootstrap) was performed at 1000 replicates, and the phylogenetic relationship between analyzed sequences was considered reliable when the bootstrap value was ≥ 70%. The obtained sequence was deposited in the GenBank under accession number MK347428.

## Results and Discussion

**Fig. 2:**
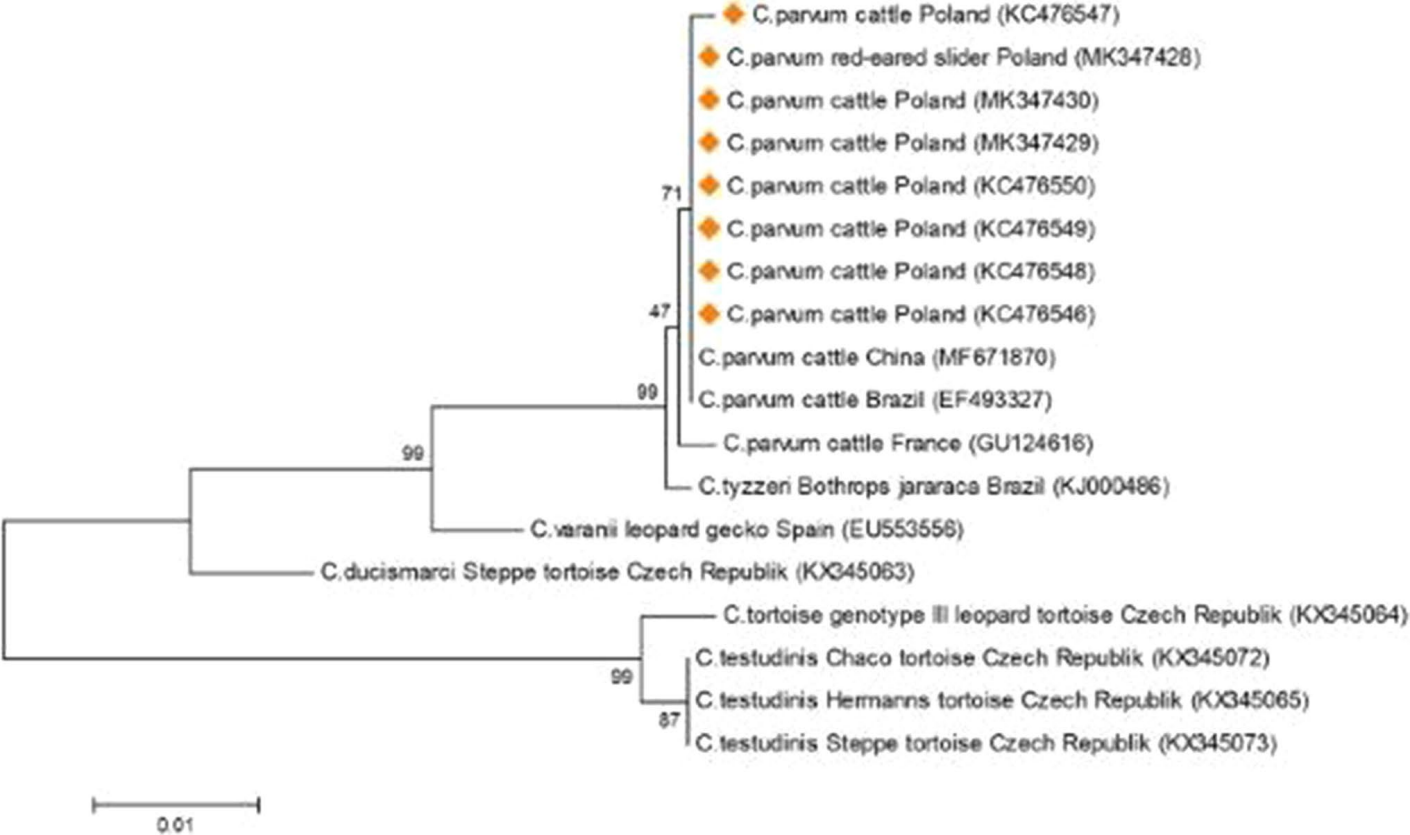
The phylogenetic tree constructed with a neighbour-joining method with Kimura two-parameter model (MEGA 7.0.9) using the nucleotide sequences (693 bp) of the 18SSU rRNA gene fragment of strains *Cryptosporidium* tortoise genotype III, *Cryptosporidium varanii*, *C. ducismarci*, *C. tyzzeri*, *C. testudinis* and *C. parvum* strains detected in red-eared slider in this study and cattle. *C. parvum* strains identified in Poland were marked with orange squares

IAS encompass different animal and plant species introduced to a habitat foreign to them. They can alter the organisation and functioning of local ecosystems and be a source or mechanical carriers for different animal and human pathogens [17, 18]. Polish and European Union legislation listed the following species of turtles as IAS: *Trachemys scripta* (red-eared slider, Cumberland slider, and yellow-bellied slider), *Chelydra serpentina* (common snapping turtle), *Chrysemys picta* (painted turtle) and *Graptemys pseudogographica* (false map turtle) [19]. In this study a molecular survey was conducted aiming to detect *Cryptosporidium* infections in IAS turtles captured from freshwater ecosystems of the Lublin province. All the animals trapped for this research were in good condition without any symptoms of systematic disease except one weakened red-eared slider turtle with anemia and swollen eyelids (personal communication with Dr Aleksandra Maluta and Dr Nadia Chlebicka). Although, other turtles did not show any symptoms of disease, in some animals, the macroscopic lesions were observed in internal organs during necroscopy. The presence of *Cryptosporidium* DNA was only detected in one sample of intestinal scraping collected from a red-eared slider which was found in Uściwierz Lake in Polesie National Park. Subsequent 18SSU rRNA sequence analysis revealed the presence of *C. parvum* (GenBank No. MK347428). The captured male turtle weighed 540 g and was in good health without signs of any systematic disease. A physical examination revealed the presence of rachitic changes in the carapace with confined losses in the stratum corneum of the plastron. At necropsy there were no changes observed in the pleuro-peritoneal cavity except the enlargement of the liver and gallbladder. There were no signs of gastroenteritis. The COWP-PCR analysis gave a negative result but an insufficient amount of DNA sample was available for subsequent GP60 subtyping. The phylogenetic analysis of the 18SSU rRNA gene fragment showed 100% sequence identity between the *C. parvum* strain isolated from the turtle and other *C. parvum* strains previously detected in cattle from Lublin province. A neighbour-joining analysis also indicated that all *C. parvum* strains of cattle origin found in Poland, France, China and Brazil are related and share 99.5–100% nucleotide sequence identity (Fig. 2). Although strain identification at sub-genotype level was not successful, the observed sequence similarities between strains and their subtype assignment strengthen the assumption that the turtle *C. parvum* strain could be of livestock origin. There was no evidence that the wild-living turtle was truly infected rather than merely passing oocysts through its gastrointestinal tract and acting only as a mechanical parasite carrier. The lack of clinical signs of infection or anatomopathological changes in its gastrointestinal tract may support this assumption. It has previously been shown that environmental abundance of *C. parvum* oocysts in areas of high livestock production led to contamination of fresh produce grown in these areas [20]. In this study, one possibility is that oocyst transmission to the turtle was via *Cryptosporidium*-contaminated water, but another is acquisition of the parasite through contact with cattle manure used as a crop fertiliser.

The majority of reports dealing with reptile cryptosporidiosis concern snakes, lizards, iguanas or tortoises kept in captivity [21–23]. They usually describe fatal cases of infections characterised by enteritis in which parasites were observed microscopically in the intestinal content [3, 5]. However, one study found that while *Cryptosporidium* contributed significantly to animal death, it was not solely responsible [5]. Usually infections in reptiles have an asymptomatic course and disease is triggered by an environmental stressor. As with wild-living tortoises, *Cryptosporidium* infections in turtles are not well documented, but *Cryptosporidium* oocysts were identified in fecal and intestinal contents of free-ranging marine green turtles (*Chelonia mydas*) and in bog turtles (*Clemmys muhlenbergi*) [7, 8].

Here, molecular tools were used to study *Cryptosporidium* prevalence in wild turtles with methods more sensitive than microscopy. Of note is that there is no standardization for protocols used for the detection of oocysts in animal feces or to diagnose the infection. Nevertheless, the reliability of the primer sets targeting *Cryptosporidium* 18SSU rRNA and COWP gene fragments for specific parasite detection has previously been proven [14]. It is noteworthy that cryptosporidial 18SSU rRNA protocols are more sensitive, therefore they are mainly recommended for the detection of parasite DNA in fecal samples of different animal species [15, 24]. Despite this, only a sporadic occurrence of *C. parvum* in wild turtles was found. Infrequent detection of this protozoan parasite in the studied turtle population could be associated with a low occurrence of infections. Although feces were collected from majority of captured turtles for an assessment of infection occurrence, the intestinal scrapings were also sampled. However, they were collected after 14 days of a quarantine period when oocysts shedding might not occur due to parasite clearance. This could be considered as a major limitation of the study. Nevertheless, one sample of intestinal scrapings was positive for *Cryptosporidium* DNA, although feces collected from this animal was parasite-free. It is not surprising, as an intermittent *Cryptosporidium* shedding is often observed, therefore testing of single fecal sample after animal capture did not guarantee a detection of infection. It would be highly valuable to test a pair of fecal samples few days (weeks) apart to obtain more accurate data. However, it will be very difficult to meet this condition for captured wildlife. Nevertheless, this approach was partly addressed by testing feces and intestinal scrapings at least for some animals. Other infectious disease co-occurrence is usual when reptile cryptosporidiosis is observed or it affects reptiles being kept in stressful environmental conditions which quickly deteriorate their health resulting in death. Of note, most of the captured and examined turtles were healthy without any symptoms of disease. Their good health status was also confirmed by necroscopy (results not shown in this study). In this light, the sporadic discovery of *C. parvum* in IAS turtles may indicate that they could play a minor role in transmission of zoonotic *Cryptosporidium* to different hosts. It is highly likely that the infected turtle served only as passive carrier of this protozoan parasite. Nevertheless, these data could be useful in order to determine the public health risk related to pet turtles of IAS species as a source of zoonotic *Cryptosporidium* in general, and in particular during an epidemiological investigation of disease cases in humans. Meanwhile, the results provide evidence for possible transmission of zoonotic *Cryptosporidium* species by IAS turtles.
